# Promoting psychological crisis coping through physical activity: The mediating roles of rumination and emotion regulation among college students

**DOI:** 10.1371/journal.pone.0350928

**Published:** 2026-06-03

**Authors:** Yang Yang, Bochun Lu, Shimeng Wang

**Affiliations:** Institute of Sports Science, Nantong University, Nantong, China; Fakultet za pravne i poslovne studije dr Lazar Vrkatic, SERBIA

## Abstract

This study examined the associations between physical activity and crisis coping among college students, with a particular focus on the potential mediating roles of rumination and emotion regulation strategies, including expressive suppression and cognitive reappraisal. A total of 1,033 Chinese college students (615 males, 418 females; aged 17–23 years, M = 20.30, SD = 1.59) completed self-report questionnaires. Structural equation modeling was used to test the proposed mediation and chain mediation models. The results indicated that physical activity was associated with both adaptive and maladaptive dimensions of crisis coping. In addition, significant indirect associations were observed through rumination, expressive suppression, and cognitive reappraisal. Two chain-mediated pathways involving rumination and emotion regulation strategies were also supported. Together, these findings suggest that rumination and emotion regulation are relevant psychological processes linking physical activity with crisis coping. This study contributes to a more nuanced understanding of how physical activity relates to crisis coping by highlighting the roles of cognitive and emotional regulation processes. The findings may inform the development of physical activity–based approaches that consider both cognitive and emotional factors in supporting college students’ mental health.

## Introduction

Contemporary college students are at a critical developmental stage marked by rapid physical, psychological, and social changes. During this period, they are exposed to a wide range of stressors, including academic demands, interpersonal difficulties, emotional challenges, and uncertainty about future careers, all of which place considerable strain on psychological adjustment [1]. Psychological crises—commonly defined as acute disruptions in cognitive and emotional regulation in response to overwhelming stress—are often accompanied by intense emotional reactions and functional impairment and may have lasting consequences for personality development, self-regulatory capacity, and long-term social adaptation [2,3]. In recent years, mental health problems among college students have become increasingly prevalent, with earlier onset and greater concealment, while traditional support systems such as campus counseling services and classroom-based mental health education have struggled to meet students’ diverse needs [4]. The post-pandemic context has further intensified these challenges, as prolonged uncertainty, social isolation, and disrupted daily routines have weakened many students’ existing coping resources [5]. As a result, identifying scalable, sustainable, and accessible approaches to strengthen crisis coping capacity has emerged as a pressing issue in contemporary mental health and public health research, particularly within the context of Chinese higher education, where students face intense academic competition, employment pressure, and ongoing sociocultural change [6].

Physical activity (PA) has been increasingly recognized as a promising health behavior with psychological benefits that extend beyond physical fitness. Research has shown that regular physical activity is associated with regulatory effects across behavioral, cognitive, and emotional domains [7]. At the physiological level, exercise supports neuroplastic processes and neurotransmitter regulation, including increases in dopamine, serotonin, and brain-derived neurotrophic factor (BDNF), which are known to contribute to stress resilience [8]. At the psychological level, physical activity has been described as a form of “psychological immune function,” enabling individuals to mobilize internal resources when confronting stress or crisis [9]. Despite this growing body of evidence, much of the existing research has focused on overall associations between physical activity and mental health or has relied on composite indicators of coping. As a result, the specific cognitive and emotional processes through which physical activity influences crisis coping remain insufficiently understood.

Another limitation of the current literature lies in how crisis coping itself is conceptualized. Contemporary coping theories consistently emphasize that coping is not a single, uniform capacity but a multidimensional process [10]. In crisis situations, individuals often draw on a mixture of coping strategies, including adaptive approaches such as problem solving and rationalization, as well as maladaptive responses such as avoidance, self-blame, and fantasy thinking, which are more strongly associated with adverse psychological outcomes [11,12]. When these diverse strategies are combined into a single composite score, important psychological distinctions may be lost, as different coping responses may relate to cognitive and emotional mechanisms in fundamentally different ways [13]. For this reason, greater conceptual clarity can be achieved by examining crisis coping as at least two theoretically distinct dimensions: adaptive and maladaptive coping.

Within this multidimensional perspective, rumination and emotion regulation emerge as two closely related mechanisms that may help explain how physical activity influences crisis coping. Rumination involves repetitive, passive attention to negative emotional experiences and their causes and has been widely identified as a transdiagnostic vulnerability factor linked to impaired problem solving, reduced cognitive flexibility, and less effective coping under stress [14,15]. Emotion regulation strategies, particularly cognitive reappraisal and expressive suppression, also play a central role in shaping responses to crisis situations [16]. Cognitive reappraisal has been consistently associated with psychological resilience and more effective recovery from stress, whereas habitual reliance on expressive suppression has been linked to emotional exhaustion and maladaptive psychological outcomes [17,18]. Importantly, these two processes are not independent. Individuals who engage in higher levels of rumination are more likely to rely on maladaptive regulation strategies, whereas effective use of cognitive reappraisal can disrupt ruminative cycles and support emotional recovery [19,20]. Emerging evidence further suggests that physically active individuals tend to report lower levels of rumination, greater use of cognitive reappraisal, and less reliance on expressive suppression when coping with stress [21–23].

**Fig 1:**
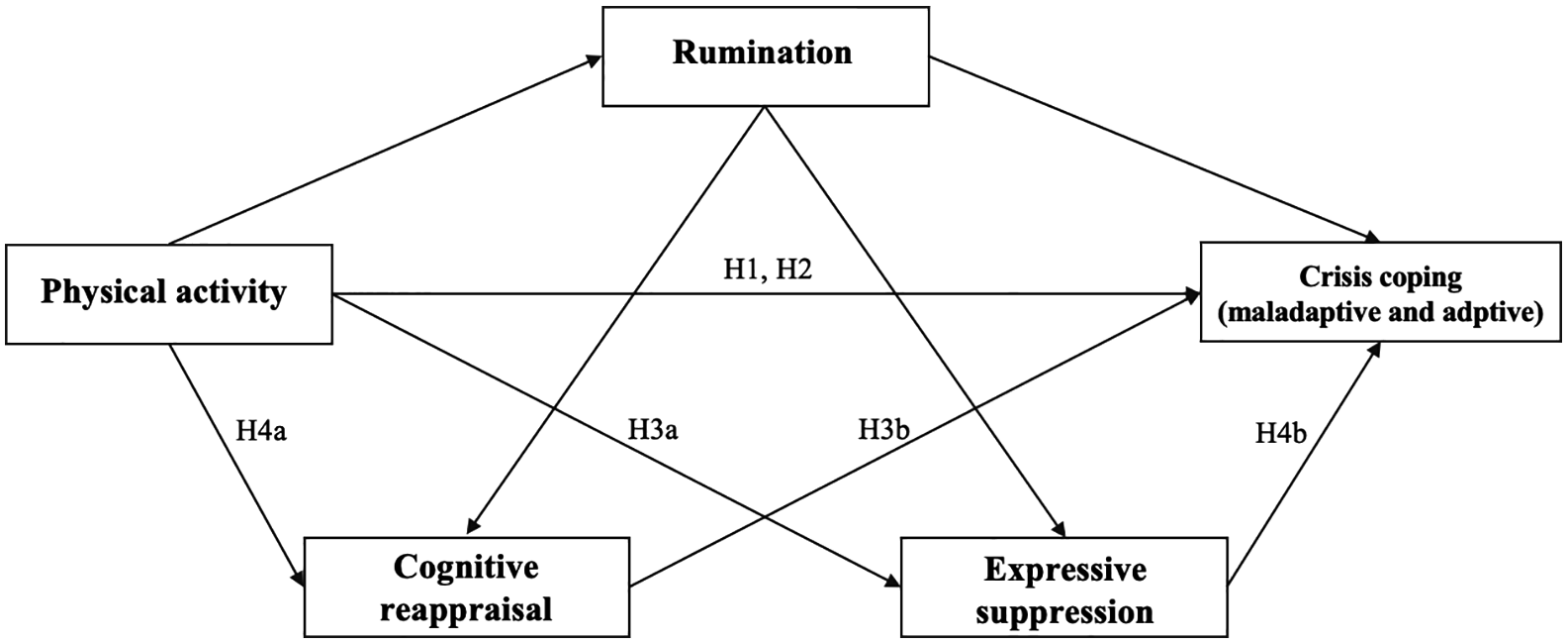
Proposed structural model illustrating the direct and indirect effects of physical activity on crisis coping through rumination and emotion regulation strategies. (Crisis coping was conceptualized as a two-dimensional construct comprising adaptive and maladaptive coping, which were tested in separate structural models).

Building on stress–coping theory [24], emotion regulation theory [16], and cognitive–behavioral perspectives, the present study proposes a structural model to examine the effects of physical activity on crisis coping among college students. Crisis coping is conceptualized as a two-dimensional construct comprising adaptive and maladaptive coping strategies, which are examined in separate structural models (Fig 1). Specifically, this study investigates the direct associations between physical activity and both dimensions of crisis coping, as well as the mediating and chain-mediating roles of rumination and emotion regulation strategies (cognitive reappraisal and expressive suppression). By clarifying the multidimensional nature of crisis coping and specifying the sequence of cognitive and emotional processes involved, this research aims to refine theoretical understanding and provide evidence relevant to the development of targeted, behavior-based mental health interventions for college students.

Based on the proposed structural model, the present study formulated the following hypotheses. H1: Physical activity is significantly associated with adaptive and maladaptive crisis coping. H2: Rumination mediates the relationship between physical activity and both dimensions of crisis coping. H3a: Cognitive reappraisal mediates the relationship between physical activity and crisis coping. H3b: Expressive suppression mediates the relationship between physical activity and crisis coping. H4a: Rumination and cognitive reappraisal jointly mediate the relationship between physical activity and crisis coping (chain mediation). H4b: Rumination and expressive suppression jointly mediate the relationship between physical activity and crisis coping (chain mediation). Note: Crisis coping is conceptualized as a two-dimensional construct comprising adaptive and maladaptive coping strategies, which were examined in separate structural models.

## Methods

### Ethics approval and informed consent

This study was approved by the Ethics Committee of Nantong University (Approval No.: TD-2024–109). Before the online questionnaire began, trained staff provided participants with a brief explanation of the study aims and procedures, either on site or by telephone. Participants then reviewed the electronic information sheet displayed on the first page of the survey. Participation required electronic written consent. Respondents could proceed only after selecting an “I agree to participate” checkbox. The survey platform recorded time-stamped consent and prevented access if consent was not provided. For participants under 18 years of age, electronic written consent was obtained from parents or legal guardians. Guardians were contacted on site or via WeChat, and consent was submitted electronically and time-stamped by the system before participation. Minors also provided electronic assent by selecting a separate assent checkbox. No direct personal identifiers were collected. All data were de-identified and analyzed in aggregate form.

### Participants

A convenience sampling strategy was used in this study. The questionnaire was created using the Wenjuanxing platform (https://www.wjx.cn) and distributed through common Chinese social media platforms, including WeChat and QQ. Recruitment took place across universities in Jiangsu, Anhui, and Liaoning provinces. Because senior students are often occupied with graduation-related requirements and may have limited availability, participation was restricted to first- to third-year undergraduate students. Data collection lasted four weeks, from April 16 to May 16, 2025. A total of 1,157 questionnaires were returned. Responses were excluded if more than 30% of items were missing, if gender or age information was not provided, or if identical or patterned responses were observed across items. For questionnaires with less than 30% missing responses, missing values were handled using listwise deletion in subsequent analyses. After applying these criteria, 1,033 questionnaires were retained for analysis.. Of the valid participants, 615 were male (59.5%) and 418 were female (40.5%). Ages ranged from 17 to 23 years, with a mean age of 20.30 years (SD = 1.59).

### Measures

#### Physical activity.

In this study, the Physical Activity Rating Scale (PARS-3), revised by [25], was employed to assess the level of physical activity among students. The scale comprises three items that measure the intensity (e.g., “What is the usual intensity of your physical activity?”), duration (e.g., “How long does each session of physical activity last?”), and frequency (e.g., “How often do you engage in physical activity per month/week?”) of physical activity. Each item provides five response options, rated on a scale from 1 to 5. For example, the options for physical activity frequency range from “less than once a month” (1 point) to “daily” (5 points). The total physical activity score is calculated using the following formula: Physical Activity Score = Intensity × (Duration – 1) × Frequency. The score ranges from 0 to 100, with physical activity levels categorized into three groups: low (≤ 19), moderate (20–42), and high (≥ 43). In the present study, the PARS-3 demonstrated good internal consistency, with a Cronbach’s α coefficient of 0.746.

#### Rumination.

The Rumination Response Scale (RRS) was originally developed by Nolen-Hoeksema and later revised into a Chinese version by Han and Yang [26]. The scale consists of 22 items and uses a 4-point Likert rating system, with higher scores indicating a stronger tendency toward rumination. The RRS includes three dimensions: Symptomatic Rumination, Compulsive Meditation, and Reflective Pondering. Specifically, Symptomatic Rumination comprises items 1, 2, 3, 4, 6, 8, 9, 14, 17, 18, 19, and 22; Compulsive Meditation includes items 5, 10, 13, 15, and 16; and Reflective Pondering covers items 7, 11, 12, 20, and 21. The Chinese version of the RRS has been widely validated among Chinese college students and has demonstrated good structural validity and reliability [26]. Previous research has shown that the RRS is an effective instrument for assessing rumination tendencies in university populations. In this study, the RRS exhibited high internal consistency, with a Cronbach’s α coefficient of 0.935.

#### Crisis coping.

In this study, a subscale from the College Students’ Stress Response Questionnaire developed by Duan [27] was employed to measure coping strategies in response to psychological crises among college students. This instrument comprises 32 items encompassing five dimensions: problem solving, fantasy thinking, avoidance, self-blame, and rationalization. It captures the specific behavioral and cognitive strategies individuals adopt when encountering psychological crisis situations. Responses are rated on a 5-point Likert scale, with higher scores reflecting greater frequency of strategy use. Following prior conceptualizations, problem solving and rationalization were classified as adaptive crisis coping strategies, whereas fantasy thinking, avoidance, and self-blame were categorized as maladaptive crisis coping strategies. In the current sample, the scale demonstrated excellent internal consistency, with a Cronbach’s alpha of 0.956, indicating strong reliability and measurement stability.

#### Emotion regulation.

This study employed the Emotion Regulation Questionnaire (ERQ), developed by Gross and John [28], to assess college students’ emotion regulation strategies. The Chinese version of the scale was adapted by Wang et al. [29] and has demonstrated good reliability and validity among Chinese adolescents. The cognitive reappraisal dimension includes seven items, which assess the tendency to regulate emotions by changing the way one thinks about emotion-eliciting situations. The expressive suppression dimension also consists of seven items, measuring the extent to which individuals inhibit the outward expression of their emotions after experiencing them. All items are rated on a 7-point Likert scale, with higher scores indicating more frequent use of the respective strategy. In this study, the Cronbach’s α coefficient for the cognitive reappraisal dimension was 0.893, and for the expressive suppression dimension was 0.889, indicating high internal consistency of the scale within the current sample.

#### Measurement model evaluation.

The reliability and convergent validity of the measurement model were assessed using Cronbach’s α, composite reliability (CR), and average variance extracted (AVE). All standardized factor loadings were above 0.67, indicating acceptable convergent validity. AVE values ranged from 0.489 to 0.612, and CR values ranged from 0.819 to 0.925, exceeding the recommended threshold of 0.80. These results indicate satisfactory internal consistency and construct reliability. Although the AVE value for symptomatic rumination was slightly below the recommended criterion, its relatively high factor loadings and CR value (0.896) support its acceptable convergent validity. Taken together, the measurement model demonstrated adequate reliability and convergent validity(see Table 1).

**Table 1:** Confirmatory factor analysis results, reliability, and convergent validity.

Second-order construct	First-order construct	Item-level Standardized factor loadings (λ)	Second-order loading	AVE	CR
Rumination	Symptomatic Rumination	0.677, 0.687, 0.699, 0.708, 0.736, 0.677, 0.698, 0.708, 0.737, 0.656, 0.668, 0.752	0.823	0.495	0.921
	Reflective Pondering	0.691, 0.675, 0.714, 0.703, 0.713	0.826	0.491	0.828
	Compulsive Rumination	0.703, 0.676, 0.741, 0.750, 0.691	0.802	0.504	0.835
Maladaptive Crisis Coping	Fantasy Thinking	0.762, 0.826, 0.751, 0.814, 0.800, 0.816, 0.764	0.758	0.636	0.924
	Avoidance	0.773, 0.770, 0.756, 0.772, 0.688, 0.690	0.780	0.542	0.876
	Self-Blame	0.762, 0.826, 0.780, 0.803, 0.775, 0.771, 0.741, 0.777	0.802	0.608	0.925
Adaptive Crisis Coping	Problem Solving	0.795, 0.782, 0.813, 0.775, 0.765	0.779	0.626	0.893
	Rationalization	0.736, 0.689, 0.738, 0.743, 0.743, 0.766, 0.702	0.835	0.534	0.889
Emotion Regulation	Cognitive Reappraisal	0.728, 0.753, 0.723, 0.744, 0.773, 0.728, 0.712	–	0.537	0.89
	Expressive Suppression	0.739, 0.784, 0.739, 0.836, 0.759, 0.773, 0.770, 0.756, 0.688, 0.772, 0.690	–	0.553	0.931

Note. All values are standardized factor loadings (λ). AVE = average variance extracted; CR = composite reliability. Crisis coping was modeled as two second-order constructs (maladaptive and adaptive). All factor loadings were statistically significant (p < .001).

A second-order confirmatory factor analysis was conducted to further examine the factorial structure of the model. The model showed good overall fit to the data (χ²/df = 1.10; CFI = 0.994; TLI = 0.994; IFI = 0.994; NFI = 0.940). The RMSEA value was low (0.010), with a 90% confidence interval of [0.007, 0.012] and a PCLOSE value of 1.00. These fit indices suggest that the measurement model fits the data well and provides an adequate basis for subsequent structural equation modeling analyses.

#### Data analysis.

Structural equation modeling (SEM) was conducted using AMOS version 26.0. The sample size was considered adequate for SEM analysis, as it exceeded the commonly recommended ratio of at least ten cases per observed variable. The model included 76 observed variables, and the final analytic sample consisted of 1,033 participants, satisfying basic sample size requirements. Statistical significance was evaluated at a p value of <.05. The mediating effects were tested using the bootstrap method with 95% confidence intervals, which is recommended for assessing indirect effects in structural equation models.

## Results

### Assessment of common method bias

Because the data were collected using anonymous self-report questionnaires, the potential risk of common method bias (CMB) was considered. Following the recommendations of Zhou and Long [30], several procedural measures were applied to reduce this risk. The questionnaire was carefully designed to ensure cultural and linguistic suitability for Chinese college students. In addition, participants were informed that their responses were anonymous and confidential, which may help reduce social desirability bias. Harman’s single-factor test was then conducted in SPSS 26.0 to statistically examine the presence of CMB. An unrotated principal component analysis was performed with the number of factors fixed at one. The results showed that the first factor accounted for 25.589% of the total variance, which is below the commonly used threshold of 40% [31]. These findings suggest that common method bias is unlikely to pose a serious threat to the results of this study..

### Descriptive statistics and correlations among study variables

Preliminary analyses showed that all study variables were approximately normally distributed. Skewness values ranged from −0.53 to 0.40, and kurtosis values ranged from −0.93 to −0.71. All values fell within commonly accepted thresholds, indicating no substantial violations of normality assumptions. These results support the use of Pearson correlation analyses and structural equation modeling in subsequent analyses.

Descriptive statistics and Pearson correlation coefficients for physical activity, rumination, emotion regulation strategies, and crisis coping variables are presented in Table 2. Physical activity was negatively correlated with rumination (r = −0.316, *p* < .01) and expressive suppression (r = −0.401, *p* < .01). In contrast, physical activity showed positive correlations with cognitive reappraisal (r = 0.368, p < .01), adaptive crisis coping (r = 0.320, *p* < .01), and maladaptive crisis coping (r = 0.369, *p* < .01). Rumination was negatively associated with adaptive crisis coping and cognitive reappraisal, and positively associated with expressive suppression, suggesting a less adaptive pattern of emotional processing.

**Table 2:** Descriptive statistics and correlations among physical activity, rumination, emotion regulation strategies, and crisis coping.

Variables	M	SD	PA	MCP	ACP	RUM	CR	ES
PA	42.01	29.77	1					
MCP	3.30	0.86	.369^**^	1				
ACP	3.35	0.91	.320^**^	.752^**^	1			
RUM	2.73	0.68	−.316^**^	−.277^**^	−.253^**^	1		
CR	4.59	1.44	.368^**^	.378^**^	.331^**^	−.279^**^	1	
ES	4.44	1.47	−.401^**^	−.347^**^	−.312^**^	.357^**^	−.599^**^	1

Note: PA = physical activity; MCP = maladaptive crisis coping; ACP = adaptive crisis coping; RUM = rumination; CR = cognitive reappraisal; ES = expressive suppression.

Adaptive and maladaptive crisis coping were moderately to strongly correlated (r = 0.752, *p* < .01). This finding likely reflects the tendency for individuals to use multiple coping strategies simultaneously when facing crisis-related stress, rather than relying on a single type of coping response. Despite this association, the two coping dimensions showed different patterns of relationships with rumination and emotion regulation strategies. This supports their conceptual distinction and justifies their treatment as separate constructs in the subsequent structural analyses.

### Structural models linking physical activity to crisis coping

Structural equation modeling (SEM) was used to test the hypothesized relationships among physical activity, rumination, emotion regulation, and crisis coping. Model fit indices are reported in Table 3. Both structural models showed satisfactory overall fit to the data. For the adaptive crisis coping model, the relative chi-square value was χ²/df = 1.30. Incremental fit indices indicated good model fit (CFI = 0.985; TLI = 0.984). The RMSEA value was low (0.017), with a 90% confidence interval of [0.015, 0.019], suggesting a close fit to the data. Similar results were observed for the maladaptive crisis coping model. The model yielded a χ²/df value of 1.32, with high incremental fit indices (CFI = 0.986; TLI = 0.985). The RMSEA value was also 0.017, with a 90% confidence interval of [0.015, 0.020]. Overall, these results indicate that both structural models provide an adequate representation of the observed data and meet commonly accepted criteria for model fit.

**Table 3:** Fit indices for structural equation models of adaptive and maladaptive crisis coping.

Model	*X*^2^/df	CFI	TLI	RMSEA (90% CI)
Adaptive crisis coping model	1.302	0.985	0.984	0.017 [0.015, 0.019]
Maladaptive crisis coping model	1.315	0.986	0.985	0.017 [0.015, 0.020]

Note. *X*²/df = relative chi-square; CFI = comparative fit index; TLI = Tucker–Lewis index; RMSEA = root mean square error of approximation.

**Fig 2:**
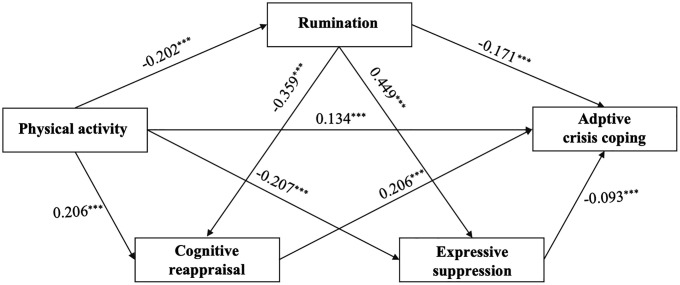
Structural model of physical activity, rumination, emotion regulation, and adaptive crisis coping.

As shown in Fig 2, physical activity was positively associated with adaptive crisis coping (β = 0.134, *p* < .001). Physical activity was also negatively associated with rumination (β= −0.202, *p* < .001). Rumination, in turn, showed a negative association with adaptive crisis coping (β= −0.171, *p* < .001), indicating an indirect pathway through rumination. In addition, physical activity was positively associated with cognitive reappraisal (β = 0.206, *p* < .001) and negatively associated with expressive suppression (β= −0.207, *p* < .001). Cognitive reappraisal was positively associated with adaptive crisis coping (β = 0.206, *p* < .001), whereas expressive suppression was negatively associated with adaptive crisis coping (β= −0.093, *p* < .001). Rumination was positively related to expressive suppression (β = 0.449, *p* < .001) and negatively related to cognitive reappraisal (β= −0.359, *p* < .001). Together, these paths supported the hypothesized indirect and chain-mediated associations between physical activity and adaptive crisis coping through rumination and emotion regulation strategies.

**Fig 3:**
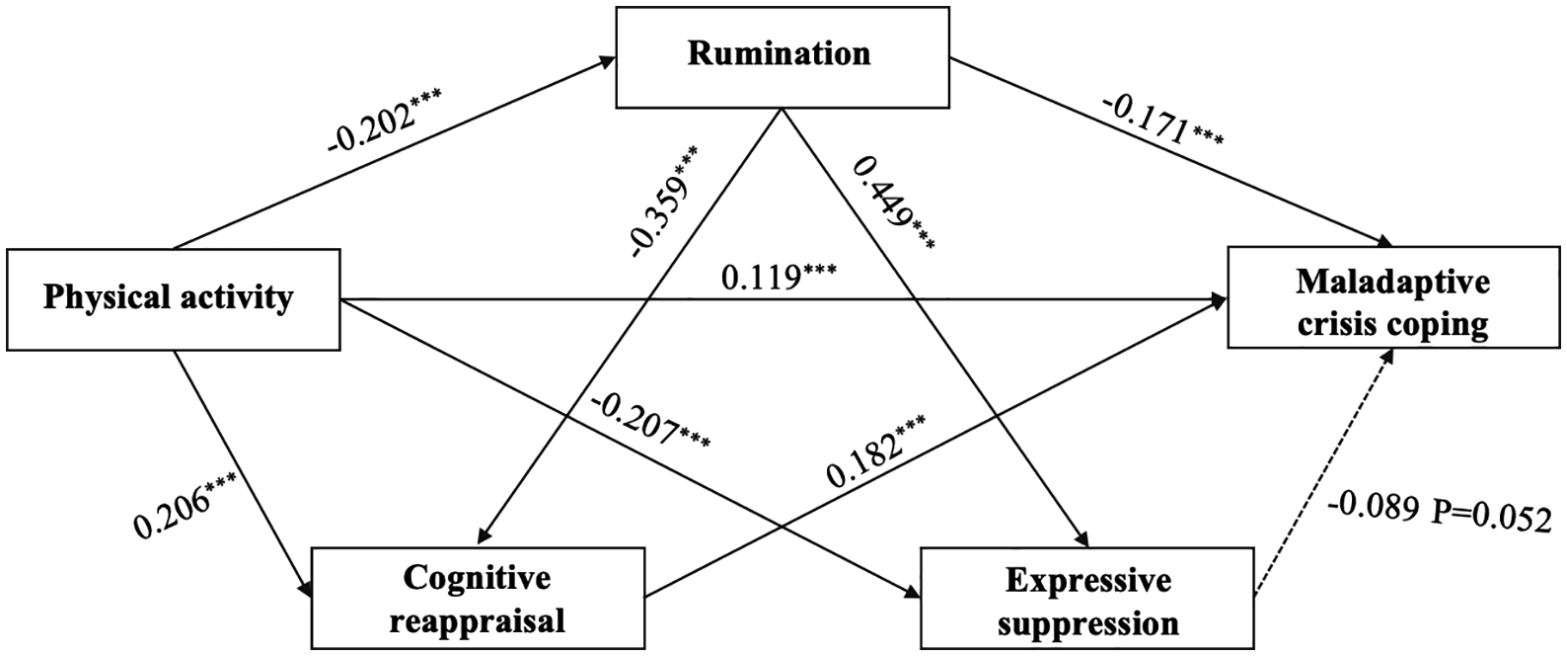
Structural model of physical activity, rumination, emotion regulation, and maladaptive crisis coping. (Standardized path coefficients are reported. Solid lines indicate significant paths; dashed lines indicate non-significant paths.).

As shown in Fig 3, physical activity was positively associated with maladaptive crisis coping (β = 0.119, *p* < .001). Physical activity was also negatively associated with rumination (β = −0.202, *p* < .001). Rumination, in turn, was positively associated with maladaptive crisis coping (β = 0.171, *p* < .001), indicating an indirect pathway through rumination. Physical activity showed a positive association with cognitive reappraisal (β = 0.206, *p* < .001) and a negative association with expressive suppression (β = −0.207, *p* < .001). Cognitive reappraisal was positively associated with maladaptive crisis coping (β = 0.182, *p* < .001). The association between expressive suppression and maladaptive crisis coping did not reach conventional levels of statistical significance (β = −0.089, *p* = .052). In addition, rumination was negatively associated with cognitive reappraisal (β = −0.359, *p* < .001) and positively associated with expressive suppression (β = 0.449, *p* < .001). Taken together, these results supported the proposed indirect and chain-mediated associations between physical activity and maladaptive crisis coping through rumination and emotion regulation strategies.

### Indirect and chain-mediated associations through rumination and emotion regulation

To examine the mediating roles of rumination and emotion regulation strategies in the relationship between physical activity and crisis coping, a bootstrapping procedure with 5,000 resamples was conducted using AMOS 26.0.

#### Results for the maladaptive crisis coping model.

As shown in Table 4, the total indirect effect of physical activity on maladaptive crisis coping was significant (β = 0.115, 95% CI [0.086, 0.146]). The direct association between physical activity and maladaptive crisis coping also remained significant (β = 0.134, 95% CI [0.088, 0.183]). Together, these effects yielded a significant total effect (β = 0.249, 95% CI [0.205, 0.295]), indicating partial mediation. Five specific indirect pathways were statistically significant. The indirect pathway through rumination alone was significant (β = 0.029, 95% CI [0.011, 0.052]), accounting for 25.22% of the total indirect effect. The pathway through cognitive reappraisal alone showed the largest contribution (β = 0.042, 95% CI [0.025, 0.064]), explaining 36.52% of the total indirect effect. The indirect pathway through expressive suppression alone was also significant (β = 0.019, 95% CI [0.004, 0.037]), contributing 16.52% of the total indirect effect. Two chain-mediated pathways were also identified. The pathway via rumination followed by cognitive reappraisal was significant (β = 0.015, 95% CI [0.008, 0.024]), accounting for 13.04% of the total indirect effect. The pathway via rumination followed by expressive suppression was likewise significant (β = 0.008, 95% CI [0.002, 0.016]), contributing 6.96% of the total indirect effect. Taken together, these results indicate that rumination and emotion regulation strategies were involved in multiple indirect pathways linking physical activity with maladaptive crisis coping.

**Table 4:** Chain mediated effects of rumination and emotion regulation on maladaptive crisis coping.

Path	Standardized indirect effect (β)	Bootstrap 95%CI		Relative inter-mediary effect%
		Lower	Upper	
physical activity → rumination → maladaptive crisis coping	0.029	0.011	0.052	25.22
physical activity → cognitive reappraisal →maladaptive crisis coping	0.042	0.025	0.064	36.52
physical activity → expressive suppression → maladaptive crisis coping	0.019	0.004	0.037	16.52
physical activity → rumination → cognitive reappraisal → maladaptive crisis coping	0.015	0.008	0.024	13.04
physical activity → rumination → expressive suppression → maladaptive crisis coping	0.008	0.002	0.016	6.96
Total indirect effect	0.115	0.086	0.146	100.00
Direct effect	0.134	0.088	0.183	–
Total effect	0.249	0.205	0.295	–

Note. Standardized indirect effects (β) were obtained from the AMOS user-defined estimates. Bootstrap confidence intervals were derived using the bias-corrected percentile method with 5,000 resamples.

#### Results for the adaptive crisis coping model.

As shown in Table 5, the total indirect effect of physical activity on adaptive crisis coping was significant (β = 0.112, 95% CI [0.080, 0.146]). The direct association between physical activity and adaptive crisis coping also remained significant (β = 0.119, 95% CI [0.066, 0.174]). Together, these effects resulted in a significant total effect (β = 0.231, 95% CI [0.184, 0.282]), indicating partial mediation. Five specific indirect pathways were statistically significant. The indirect pathway through rumination alone was significant (β = 0.035, 95% CI [0.014, 0.058]), accounting for 31.25% of the total indirect effect. The pathway through cognitive reappraisal alone was also significant (β = 0.038, 95% CI [0.020, 0.061]) and represented the largest proportion of the total indirect effect (33.93%). The indirect pathway through expressive suppression alone was significant (β = 0.019, 95% CI [0.001, 0.039]), contributing 16.96% of the total indirect effect. Two chain-mediated pathways were also identified. The pathway via rumination followed by cognitive reappraisal was significant (β = 0.013, 95% CI [0.007, 0.023]), accounting for 11.61% of the total indirect effect. Similarly, the pathway via rumination followed by expressive suppression was significant (β = 0.008, 95% CI [0.002, 0.018]), contributing 7.14% of the total indirect effect. Taken together, these results indicate that rumination and emotion regulation strategies were involved in multiple indirect pathways linking physical activity with adaptive crisis coping.

**Table 5:** Chain mediated effects of rumination and emotion regulation on adaptive crisis coping.

Path	Standardized indirect effect (β)	Bootstrap 95%CI		Relative inter-mediary effect%
		Lower	Upper	
physical activity → rumination → adaptive crisis coping	0.035	0.014	0.058	31.25
physical activity → cognitive reappraisal →adaptive crisis coping	0.038	0.020	0.061	33.93
physical activity → expressive suppression → adaptive crisis coping	0.019	0.001	0.039	16.96
physical activity → rumination → cognitive reappraisal → adaptive crisis coping	0.013	0.007	0.023	11.61
physical activity → rumination → expressive suppression → adaptive crisis coping	0.008	0.002	0.018	7.14
Total indirect effect	0.112	0.080	0.146	100.00
Direct effect	0.119	0.066	0.174	–
Total effect	0.231	0.184	0.282	–

Note. Standardized indirect effects (β) were obtained from the AMOS standardized indirect effects output. Bootstrap 95% confidence intervals were derived from bias-corrected bootstrap estimates (5,000 resamples). Relative inter-mediary effects were calculated as the proportion of each specific indirect effect relative to the total indirect effect.

## Discussion

### Associations between physical activity and crisis coping

The present study found that physical activity was directly associated with both adaptive and maladaptive dimensions of crisis coping. This result suggests that physical activity plays a general role in how college students respond to psychological crises, rather than influencing only a specific type of coping behavior. In crisis situations, coping responses are rarely selected in a simple adaptive–maladaptive dichotomy. Instead, individuals often engage in multiple coping strategies simultaneously in an attempt to manage intense and uncontrollable stressors. Previous coping research has similarly shown that adaptive and maladaptive strategies frequently coexist under high levels of stress [13,12].

From this perspective, the positive association between physical activity and maladaptive coping should not be interpreted as evidence of a harmful effect. Rather, it may reflect greater overall coping engagement and behavioral activation among physically active individuals when facing crisis-related stress. This interpretation is consistent with prior findings indicating that higher levels of physical activity are generally linked to better psychological functioning, including lower rates of anxiety and depression and greater tolerance of stress [32,33]. Physical activity may contribute directly to crisis coping through several complementary mechanisms. Regular exercise is known to stabilize emotional responses, reduce emotional reactivity, and promote positive affect, which can buffer the emotional intensity of crisis situations [34,35]. In addition, physical activity has been associated with improvements in executive functioning and cognitive control, allowing individuals to evaluate stressful situations more flexibly and make more adaptive decisions under pressure [36]. At a physiological level, exercise-related modulation of neuroendocrine responses, including reduced hypothalamic–pituitary–adrenal axis reactivity and increased neurotransmitter availability, may further support stress tolerance and emotional stability [8,37]. Taken together, these findings suggest that physical activity does not simply suppress maladaptive coping responses, but instead shapes the broader psychological and physiological conditions under which coping strategies are selected during crises.

### Indirect pathways through rumination and emotion regulation

Beyond these direct effects, the present findings indicate that physical activity influences crisis coping through a sequential process involving rumination and emotion regulation. Rumination appears to function as an initial cognitive mechanism in this pathway. As a repetitive and passive focus on negative emotional experiences, rumination has been widely identified as a transdiagnostic risk factor that interferes with cognitive flexibility and undermines effective coping under stress [14,15]. In line with previous experimental and intervention studies, higher levels of physical activity in the present study were associated with lower levels of rumination, supporting the idea that physical activity can disrupt ruminative thinking by redirecting attention and increasing cognitive engagement [22,23,38].

Importantly, reductions in rumination were associated with subsequent differences in emotion regulation strategy use. Individuals with lower ruminative tendencies were less likely to rely on expressive suppression and more likely to use cognitive reappraisal. Prior research has consistently shown that rumination is linked to greater use of suppression and reduced use of reappraisal, suggesting that ruminative cognition constrains flexible emotional processing [19,39]. Rumination and expressive suppression may reinforce one another: persistent rumination intensifies emotional distress, increasing the likelihood of suppressing emotional expression, while suppression limits emotional processing and prolongs ruminative thought [40,41]. The present findings suggest that physical activity may interrupt this maladaptive cycle by first reducing ruminative thinking, which in turn lowers reliance on suppression-based regulation.

At the same time, reduced rumination may facilitate the use of cognitive reappraisal by freeing cognitive and emotional resources that are otherwise consumed by repetitive negative thinking. Cognitive reappraisal requires attentional control and cognitive flexibility, capacities that are often impaired by rumination but may recover as ruminative processing decreases [42,43]. Consistent with this interpretation, previous studies have shown that individuals who engage in higher levels of physical activity are more likely to adopt adaptive reappraisal strategies when coping with stress [21]. Emotion regulation may therefore operate as a downstream mechanism through which changes in ruminative cognition are translated into observable coping behaviors.

Overall, the present results suggest that the psychological benefits of physical activity in crisis contexts are best understood as a sequential cognitive–emotional process. Rather than exerting isolated effects on specific coping strategies, physical activity appears to influence how individuals process negative experiences, which then shapes emotion regulation and, ultimately, coping responses. This chain mediation pattern provides a more nuanced account of how physical activity contributes to both adaptive and maladaptive dimensions of crisis coping among college students.

## Conclusion

This study examined the associations between physical activity and crisis coping among college students, with attention to both direct relationships and related psychological processes. The findings suggest that physical activity is associated with more adaptive coping responses, as well as with patterns of cognitive and emotional regulation relevant to how individuals respond to crisis-related stress. These associations cannot be explained by participation frequency or intensity alone. Instead, physical activity appears to be linked to internal regulatory processes, including lower levels of rumination and greater use of flexible emotion regulation strategies. From this perspective, physical activity may be viewed as a behavioral context associated with reduced engagement in repetitive negative thinking and more balanced emotional responses during periods of psychological strain. These findings have practical implications for mental health promotion among college students, indicating that physical activity–based approaches may be most valuable when they support cognitive and emotional regulation alongside regular participation.

### Limitations and future research directions

Despite the contributions of the present study in examining the associations among physical activity, rumination, emotion regulation, and crisis coping, several limitations should be acknowledged. First, the cross-sectional design restricts the ability to draw causal inferences among the variables. Although the theoretical framework and statistical models provide preliminary support for multi-path psychological relationships, longitudinal or experimental studies are needed to further examine the temporal ordering of these processes. Second, the sample consisted primarily of college students from eastern China, which may limit the generalizability of the findings. Future research could include more diverse populations, such as adolescents, working adults, or cross-cultural samples, to enhance external validity. Third, the present study focused on two commonly examined emotion regulation strategies—expressive suppression and cognitive reappraisal—which may not fully capture the complexity of regulatory processes involved in crisis coping. Other mechanisms, such as acceptance, self-compassion, or attentional processes, warrant consideration in future research. Finally, the study relied primarily on self-report measures. Although the instruments demonstrated satisfactory reliability and validity, responses may still be influenced by social desirability or subjective bias. Future studies could benefit from incorporating objective behavioral indicators (e.g., activity tracking data), psychophysiological measures (e.g., heart rate variability or EEG), and qualitative approaches to provide a more comprehensive understanding.

## Supporting information

S1 File Data. (XLSX)
